# Choroidal vascularity index and choroidal thickness: potential biomarkers in retinitis pigmentosa

**DOI:** 10.1038/s41433-022-02270-5

**Published:** 2022-10-07

**Authors:** Solmaz Abdolrahimzadeh, Mariachiara Di Pippo, Chiara Ciancimino, Federico Di Staso, Andrew John Lotery

**Affiliations:** 1 Ophthalmology Unit, Neurosciences, Mental Health, and Sense Organs (NESMOS) Department, Faculty of Medicine and Psychology, University of Rome Sapienza, Rome, Italy; 2 St. Andrea Hospital, Rome, Via di Grottarossa 1035/1039, Rome, 00189 Italy; 3 Clinical and Experimental Sciences, Faculty of Medicine, University of Southampton, Southampton, SO16 6YD UK

**Keywords:** Prognostic markers, Eye diseases

## Abstract

Retinitis pigmentosa (RP) is the commonest inherited retinal dystrophy. It is characterized by progressive photoreceptor degeneration and cell death and ongoing neuronal and vascular impairment. In recent years, pathophysiological alterations of the choroid have begun to be appreciated in RP. Thus, representing a potential diagnostic and therapeutic biomarker. In particular, choroidal thickness and the choroidal vascularity index can be used to understand the pathogenesis of disease and evaluate new therapeutic possibilities. Photoreceptor changes seen in eyes with RP are directly correlated to a decrease of choroidal flow, leading to a strong association between relative choroidal ischemia and visual impairment. In this review we analyse the literature on choroidal thickness and choroidal vascularity index in patients with RP and assess whether these markers may reflect progression of disease from an anatomical and functional point of view.

## Abstract (zh)

视网膜色素变性 (RP) 是最常见的遗传性视网膜营养不良。其特征是光感受器进行性退行性变性、细胞凋亡以及持续的神经元和血管损伤。近年来, RP的脉络膜病理生理变化开始得到重视。脉络膜病理生理相关的标记物是RP的潜在诊断和治疗的生物标记物。特别是, 脉络膜厚度和脉络膜血管指数可用于了解疾病的发病机制和评估新治疗的可能性。RP患者光感受器变化与脉络膜血流降低直接相关, 导致脉络膜相对缺血和视力损害之间具有显著正相关性。在该综述中, 我们分析了RP患者脉络膜厚度和脉络膜血管指数的相关文献, 并从解剖和功能角度评估这些标志物是否可以反映疾病的进展。

## Introduction

Retinitis pigmentosa (RP) is a degenerative hereditary disease of the retinal neuroepithelium characterized by progressive deterioration and death of retinal photoreceptors. It is the commonest hereditary retinal dystrophy with a prevalence of 1 per 3000–5000 newborns [1]. Histologically RP is identified by rod photoreceptor death followed by cone photoreceptor death at the more advanced stages of disease, resulting in thinning of the outer retinal layers and retinal vessels, waxy pallor of the optic nerve, and pigmentary changes of the retina [2]. Clinical diagnosis of RP is based on the presence of night blindness, reduced visual acuity, intraretinal pigmentation in the shape of bone spicules in the mid-periphery, attenuation of retinal arteries, an annular scotoma and/or narrowing of the visual field, vitreous degeneration with pigment dusting, and decreased or abolished electroretinograms [3].

RP is usually classified into three subtypes according to the inheritance model: autosomal dominant, autosomal recessive, or X-linked [4]. At least 49 different genes and several loci have been identified as responsible for non-syndromic forms of RP and the underlying genetic defect is identifiable in over 50% of cases. These genes encode components of the phototransduction cascade, proteins involved in retinol metabolism and cellular-cellular interaction, structural proteins of photoreceptors, transcription factors, intracellular transport proteins, and splicing factors [5].

Experimental and clinical studies have shown that there is an alteration of retinal and choroidal vascularization in the course of RP. Choroidal fluximetry studies demonstrate a significant alteration of choroidal haemodynamics with a qualitative and quantitative reduction in choroidal flow in a manner directly proportional to the severity of the disease [3, 5]. Whether these flow changes are caused by histopathological alterations of the disease or whether choroidal alterations are the primum movens of the retinal manifestations still needs to be clarified. In particular, studies in animal models of RP on mice, rats, and Abyssinian cats show a reduction in the choroidal circulation in response to phototoxicity with subsequent cell death of cones [6], increase in plasmatic endotheline-1 leading to choroidal vasoconstriction or reduction of choroidal flow [7], and progressive loss of capillaries of the choroid [8, 9]. These observations have been confirmed in humans by Langham et al. in 1990. These authors evaluated the ophthalmic arterial pressure and the pulsatile ocular blood flow in order to obtain the total blood flow to determine whether ocular haemodynamics were altered and whether these changes correlated with visual impairment. They concluded that relative choroidal ischemia is strongly associated with visual loss and degeneration of pigmented cells in patients with RP [3].

## Choroidal thickness and choroidal vascularity index assessment

**Fig. 1:**
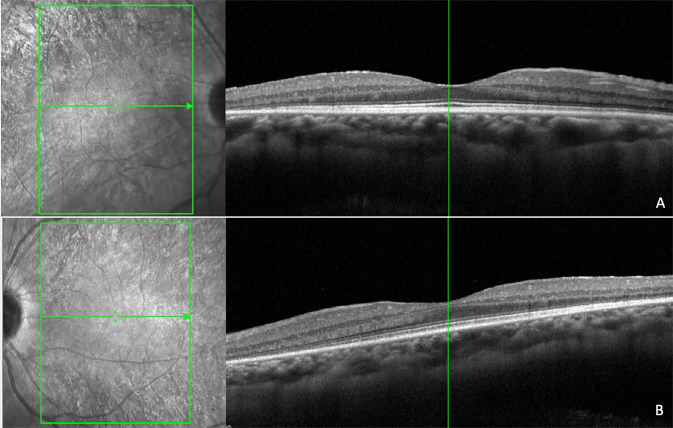
Near infrared reflectance and spectral domain optical coherence tomography scans in a patient with homozygous PDE6b mutation. There is alteration of the ellipsoid zone, interruption of the external limiting membrane, and mild hyperreflectivity of the inner limiting membrane (**A**: right eye; **B**: left eye).

**Fig. 2:**
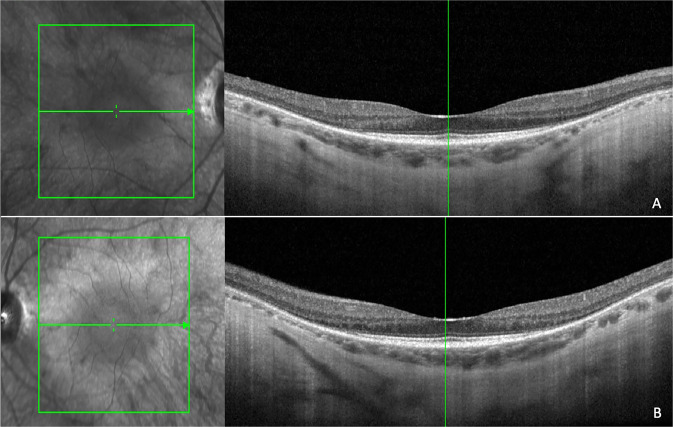
Near infrared reflectance and spectral domain optical coherence tomography scans in a myopic patient with rhodopsin rhoAsp190Asn mutation. There is alteration of the ellipsoid zone, and interruption of the external limiting membrane with choroidal thinning and structural alterations (**A**: right eye; **B**: left eye).

**Fig. 3:**
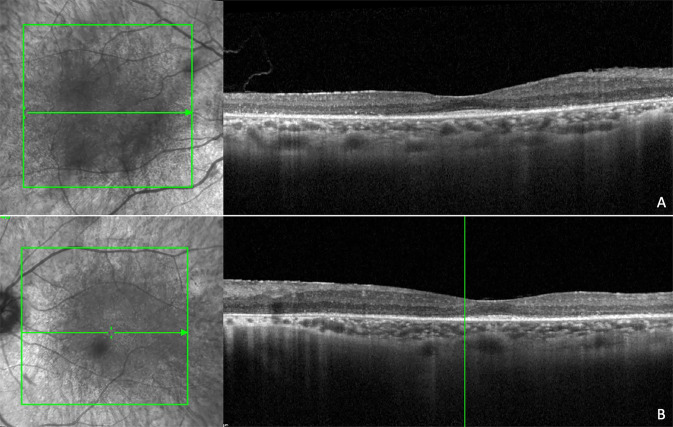
Near infrared reflectance and spectral domain optical coherence tomography scans in a patient with Usher’s disease. There are diffuse alterations of the ellipsoid zone, extensive interruptions of the external limiting membrane, hyperreflectivity of the inner limiting membrane, and disorganization of inner retinal layers. There are diffuse structural alterations of the choroidal with dot-like hyperreflectivity. (**A**: right eye; **B**: left eye).

The introduction of spectral domain optical coherence tomography (SDOCT) has enabled non-invasive and highly sensitive imaging of retinal layers [10]. SDOCT guarantees excellent focus with high resolution visualization of the retina. There is a progressive decrease of signal towards the choroidal structures resulting in a decrease of the sensitivity and the resolution of images moving away from the zero-delay point (which corresponds to the inner retinal edge). There is a limitation of the dynamic range offered by the analogue-to-digital conversion before the transformation of Fourier and the wavelength-dependent light scattering induces a reduction of the signal-noise contribution in the information coming from the lower retinal layers. An accurate study of the choroid is possible performing SDOCT with enhanced depth imaging (EDI) technology where the scan is closer to the eye of the patient in order to obtain an inverted image showing the deeper retinal layers and the choroid closer to the zero-delay point. This permits an enhanced and high-resolution image of the choroid up to the inner portions of the sclera [11, 12]. Therefore, information on retinal architecture can be integrated with that on choroidal vasculature to better understand the pathogenesis of RP [13] (Figs. 1–3).

**Fig. 4:**
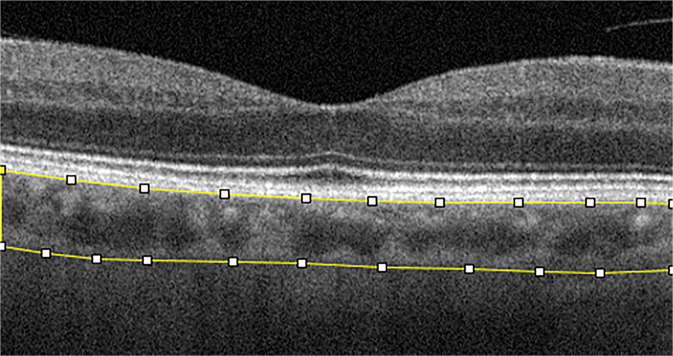
Spectral domain optical coherence tomography scan showing the region of interest. The upper limit is at the inferior margin of the retinal pigmented epithelium and the lower limit is at the choroidoscleral junction (yellow lines).

**Fig. 5:**
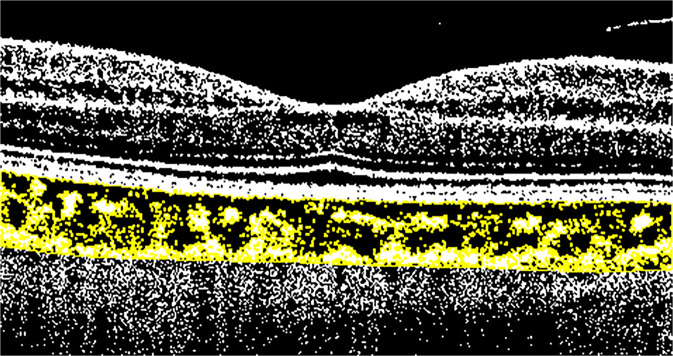
Post-acquisition spectral domain optical coherence tomography image processing showing binarization to calculate the choroidal vascularity index. Binarization is made on the region of interest (ROI) using Niblack’s auto local threshold technique, to analyze mean and standard deviation of dark and light pixels in the ROI.

One of the first choroidal parameters studied was choroidal thickness (CT). CT is a biomarker in numerous chorio-retinal diseases including central serous chorioretinopathy, age-related macular degeneration, and diabetic retinopathy [14–17]. Measurement of CT is preferably carried out on EDI scans from the inner edge of Bruch’s membrane to the choroid-scleral junction by manual measurement at the sub-fovea and in the nasal and temporal region at intervals of 500 micrometers up to 1500 micrometers from the fovea. Yamashita et al. validated this method on a sample of healthy patients assessing the repeatability of measurements using several commercially available SDOCT devices [18]. Later, mapping using a semi-manual method were also introduced to better evaluate CT [19, 20]. However, despite the validity of this marker and its relative ease of calculation, various studies showed variability of results within comparable populations, as thickness is influenced by age. Hence, the need to use an additional parameter to study the choroid in vivo. The choroid is formed by blood vessels and stroma, composed of connective tissue, melanocytes, nerves, and extracellular fluid. The choriocapillaris, is the innermost layer and guarantees the vascular and nutritive support to the outer retinal layers, Sattler’s layer is the intermediate layer composed of choroidal vessels of small and medium calibre, and Haller’s layer is the outermost layer consisting of large-calibre choroidal vessels. Given the predominantly vascular nature of the choroid, it is easy to understand that it is fundamental to study the flow and vascular distribution, as well as anatomical structure. Sonoda et al. [21] described a new technique to calculate and differentiate the choroidal stromal area from the choroidal luminal area (LA). This method is based on post-acquisition image processing using imaging binarization on open-access software. Briefly, image binarization is made with the open-source software ImageJ (distributed by Fiji, https://imagej.net/Fiji/Downloads) on horizontal EDI scans centered on the fovea by selecting a 3-millimeters choroidal area beneath the fovea. After setting the image scale, with the polygon tool of the software, a region of interest (ROI) is selected that has its upper limit at the inferior margin of the retinal pigmented epithelium and its lower limit at the choroidoscleral junction (Fig. 4). Three choroidal vessels larger than 100 microns are selected using the oval selection tool, in order to obtain the mean vascular brightness. Successively, binarization is made on the ROI using the Niblack’s auto local threshold technique, to analyse mean and standard deviation of dark and light pixels in the ROI. The software analysis system identifies areas with dark pixels, the totality of which gives the LA, while the totality of pixels in the ROI defines the total area (TA) (Fig. 5) [21]. Agrawal et al. proposed a quantitative parameter called choroidal vascularity index (CVI) that enables the assessment of choroidal vascularization with high reliability and lower variability than CT [22]. The authors adopted the method proposed previously by Sonoda et al. [21] with minor modifications (e.g., selection of polygonal area after image binarization) to calculate LA, choroidal stromal area, and total choroidal tissue. The ratio between LA and total choroidal area is the CVI [22].

Nevertheless, it must be kept in mind that the limitation of SDOCT is that functional features are inferred from morphological features.

## CT and CVI in retinitis pigmentosa

Several studies have described the choroidal changes in RP patients compared to healthy controls. Akay et al. found a reduction in CT in patients with early RP [23]. Aknin et al. found a 40% reduction of CT in patients with RP compared to healthy controls. This was directly proportional to a decrease of best corrected visual acuity (BCVA). The authors explained this association by interpreting worsening of vision as a sign of disease progression; there was gradual thinning of the choroid with advancing disease [24]. Similarly Ayton et al. conducted a study to compare the CT of patients with RP with that of a control group and to define predictors of CT in patients with RP. They also found that CT was reduced in patients with RP compared to a control group. This was directly proportional to worsening of visual acuity. Interestingly, they found that a decrease in CT was directly proportional to the duration of the disease, despite the age of patients. However, even in the more advanced stages of disease they observed a relative preservation of choroidal structure especially in the subfoveal region. This could be an important consideration in the decision-making phase of a therapeutic approach for these patients and in the evaluation of the outcome after a retinal or a suprachoroidal prosthesis implant [25]. Other results were found in a case-control study conducted by Dhoot et al. on 21 patients with RP and 25 age-matched subjects, showing a reduction in CT in RP patients. Nevertheless, the authors did not find a relationship between visual acuity and CT in the correlation analysis between these two variables and hypothesized that the absence of correlation between choroidal thinning and visual impairment is because photoreceptor damage occurs after choroidal involvement. However, the cross-sectional nature of the study represented a limit and longitudinal evaluation is required to better evaluate the relationship between CT and visual acuity over the time [26].

These findings were confirmed by Son et al., as part of a broader assessment aimed at correlating retinal and choroidal structural data with functional data in patients with RP compared to controls. In this study, in addition to a reduction in CT, the authors reported a negative correlation between CT and BCVA and age [27]. To better determine the trend of CT in relation to demographic and functional factors such as age, duration of the disability, and visual acuity, Sodi et al. conducted a study on 39 patients with RP, characterizing the stage of disease, the genetic defect, visual acuity, impairment of the visual field, response to scotopic electroretinography, and CT. The data obtained showed no correlation between CT and the clinical parameters measured, confirming, however, the reduction of the CT compared to the control group. As suggested by these authors, the lack of correspondence between the various studies on the relationship between CT and other factors such as duration of the disease is probably due to the difficulty in precisely establishing the date of onset of disease as even patients find it impossible to accurately determine the date of initiation of symptoms [28].

Unlike other authors, Chhablani et al. did not find a statistically significant difference in thicknesses in patients with RP while Tan et al. even showed an increase in CT with respect to a control group. The authors attributed these findings to the cross-sectional nature of their investigations and the lack of data concerning choroidal vascular flow, claiming the need for a parameter that would assess choroidal vascularization rather than merely structural characteristics [29, 30].

The CVI calculation is a novel method of evaluation that might meet this need and since 2019 some studies have evaluated this parameter in patients with RP. Tan et al. conducted a study with the aim to assess choroidal structural changes in patients with RP compared to healthy controls using CVI and CT as parameters. They observed a reduction in CVI in eyes with RP [30]. Wei et al. reported a similar study design in 26 patients with retinal dystrophies (17 with RP) and 32 healthy subjects. These authors found a lower mean CVI in patients with RP versus healthy controls, with no significant difference in CVI between the various groups of retinal dystrophies. The authors suggested a general tendency to reduced choroidal vascularity in all patients with retinal dystrophy and hypothesized that, in affected patients, there could be a greater propensity to atrophy of the choroidal vascular component rather than the stromal component explaining both the reduction of CVI and of CT [31].

Iovino et al. evaluated CT, central macular thickness, and CVI in the evaluation of patients with RP associated with cystoid macular oedema (CMO). A total of 159 patients with RP were included, of which 67 eyes had CMO and 92 eyes did not. The authors reported a lower CVI and a higher CT in patients with CMO. These results confirm the known role of the choroid in the aetiology of CMO and suggest the use of CVI as a possible marker of further damage in RP. In this case it was proposed that the increased CT was the result of intrastromal blood leakage due to increased vasal permeability secondary to release of inflammation mediators [32].

CVI reduction in RP was also confirmed by Shen et al. in 2020. These authors evaluated the changes in choroidal vascularity by assessing the presence of any vascular defects in the choriocapillaris and by calculating the CVI to analyse the medium/large choroidal vessel structure. They found defects in the choriocapillaris structure and CVI of medium and large choroidal vessels in the foveal, parafoveal, and perifoveal regions in RP patients, with a positive correlation between the two parameters. No correlations were found between CVI and visual acuity [33].

Cetin et al. correlated CVI with other retinal biomarkers such as alteration of the ellipsoid zone (EZ), interruption of the external limiting membrane, disorganization of the inner retinal layers (DRIL), and epiretinal membranes in patients with RP. Their results showed a positive correlation between outer retinal alterations and CVI, with an increase in CVI in the presence of DRIL or EZ interruptions. Interestingly, they found an increase of peripapillary CT, while they did not find changes in macular CT, despite an increased CVI in all sectors. In line with previous studies, no correlation of CVI with visual acuity was noted. The authors suggested that CVI variations in patients with RP probably precede structural changes of the retina and choroid, Thus, in the early stages of disease CT remains unchanged [34].

Damage in RP initiates from the peripheral retina with late involvement of the posterior pole; the hyperautofluorescent (HAF) ring represents the watershed area between the most degenerated retina, peripheral to the HAF ring, and preserved retina inside the ring. Kawano et al. evaluated choroidal changes in eyes with RP taking the HAF ring at the posterior pole as a reference. Based on this assumption, the authors evaluated the LA and the stromal choroidal area inside and outside the HAF ring. Their results showed a significant reduction in LA in RP patients compared to healthy controls and a significant reduction in LA outside the HAF ring where the retina was more profoundly involved. The stromal area however did not seem to be involved by these changes, suggesting a more prominent involvement of the LA in CVI decrease [35]. These results are consistent with that of Egawa et al. who studied the stromal and the luminal choroidal areas in patients with RP, distinguishing between the inner and outer subfoveal choroid. The secondary endpoint of their study was to correlate these results with functional and anatomical retinal changes in the course of RP. They found a significant smaller choroidal LA in the inner choroid in eyes with RP, which correlated with BCVA, foveal sensitivity, EZ width, and central foveal thickness [36]. Their results confirm those of previous studies, confirming, once again, how the LA is compromised from the earliest stages of the disease and how the relationship between the luminal and stromal components correlates with retinal damage, making CVI a reliable parameter that reflects disease progression. Table 1 is a summary of the major publications on CT and CVI.

**Table 1:** Clinical characteristics of included studies.

Study	Study design	Number of eyes (number of patients)	Primary and secondary endpoint	Results	Secondary outcomes
Akay et al. 2019 [23]	Prospective case-control	Cases: 35 (35) Controls: 40 (40)	CT in RP patients with respect to controls	Lower CT in all measurement areas	
Aknin et al. 2018 [24]	Prospective case-control	Cases: 40 (20) Controls: 40 (20)	CT in RP patients with respect to controls Association between CT and visual acuity	Lower CT in all measurement areas	Decreased visual acuity significantly associated with reduction of CT
Ayton et al. 2013 [25]	Prospective case-control	Cases: 67 (42) Controls: 35 (22)	CT in RP patients with respect to controls Association between CT, visual acuity and duration of disease.	Lower CT in all measurement areas	Reduction of CT with poorer visual acuity or longer duration of symptoms
Dhoot et al. 2013 [26]	Prospective case-control	Cases: 21 (21) Controls: 25 (25)	CT in RP patients with respect to controls Association between CT, visual acuity, and central retinal thickness	Lower CT in all measurement areas	No correlation between CT and visual acuity or central retinal thickness
Son et al. 2019 [27]	Retrospective cross-sectional	Cases: 291 (149) Controls: 68 (68)	Correlations between VFI and BCVA and structural indices (CFT, EZ length, and CT)	Lower CT in all measurement areas	No correlation between CT and VFI or BCVA
Sodi et al. 2018 [28]	Retrospective cross-sectional	Cases: 291 (39) Controls: 68 (73)	CT in RP patients respect to controls Association between CT, visual acuity, age, age at disease onset, duration, macular thickness, visual field loss, ERG	Lower CT in all measurement areas	No correlation between CT and clinical parameters
Chhablani et al. 2016 [29]	Prospective observational study	Cases: 88 (69) Controls: 188 (104)	CT in RP patients with respect to controls Association between CT, visual acuity, outer retinal structures, and age	No difference of CT in RP respect to control group	Thinning of CT with age No correlation between CT and visual acuity or clinical parameters
Tan et al. 2018 [30]	Prospective observational	Cases: 35 (35) Controls: 26 (26)	CVI and CT in RP patients with respect to controls	Lower CVI and increase in CT in RP patient	
Wei at al. 2019 [31]	Retrospective cross-sectional	Cases: 46 (26) Controls: 64 (32)	CVI in retinal dystrophies	Lower CVI in RP patient respect to controls	
Iovino et al. 2019 [32]	Retrospective cross-sectional	Cases: 159 (159) divided in two groups based on the presence or the absence of CME (resp. 67 vs 92 eyes)	CVI and CT in RP patients with and without CME	Increased CT and lower CVI in patients with CME respect to those without CME	
Shen et al. 2020 [33]	Prospective observational	Cases: 34 (63) Controls: 34 (17)	CVI and CC defects in RP patients with respect to controls	Lower CVI in RP patient respect to controls. Presence of CC defects	No correlation between CVI and visual acuity or retinal structures changes
Cetin et al. 2020 [34]	Prospective observational	Cases: 69 (36)	Study of CT, CVI, presence of DRIL, ERM and disruption of ELM and EZ in RP patients	Higher CVI in patients with disruption of outer retinal segment integrity. No correlation between CT and retinal alterations	No correlation between visual acuity and CT or CVI
Kawano et al. 2017 [35]	Retrospective cross-sectional	Cases: 37 (24) Controls: 35 (35)	Luminal area and stromal area defects in RP patients respect to controls Luminal area and stromal area inside and outside HAF ring	Luminal area reduced in RP patients respect to controls	In RP patients luminal area is reduced outside HAF respect to inside HAF
Egawa et al. 2019 [36]	Retrospective observational case series	Cases: 100 (100) Controls: 60 (60)	Luminal area and stromal area in inner and outer subfoveal choroid Correlation between luminal area and stromal area and visual acuity	Significant smaller choroidal Luminal area, in the inner choroid in eyes with RP	Correlation of luminal area with best corrected visual acuity, foveal sensitivity, EZ width, and central foveal thickness

*CT* choroidal thickness, *RP* retinitis pigmentosa, *VFI* visual field index, *BCVA* best corrected visual acuity, *CFT* central foveal thickness, *EZ* ellipsoid zone, *CVI* choroidal vascularity index, *CME* cystoid macular edema, *CC* choriocapillaris, *DRIL* disorganization of retinal inner layers, *ERM* epiretinal membrane, *ELM* external limiting membrane, *HAF* hyperautofluorescent ring.

## Conclusions

The CVI and the CT are two established parameters in the evaluation of the choroid in numerous retinal diseases. Choroid assessment in patients with RP has been the subject of study for several years. Various techniques have been employed starting from the first histopathological studies conducted ex vivo that showed loss of the choriocapillaris in parallel with retinal pigmented epithelium alterations [37], to in vivo studies evaluating hemodynamic changes in choroidal flow with doppler fluximetry and high-resolution magnetic resonance imaging techniques that showed a clear reduction in choroidal flow proportional to the progression of disease [3, 5, 38]. The role of the choroid in the pathogenetic mechanism of retinal damage in RP is not clear but it is now accepted that choroidal alterations participate as primum movens of damage. High values of plasma endotheline-1 (ET) have been found in patients with RP, probably playing an important role in the reduction of choroidal vascularization and reduction of retinal flow [39, 40]. Indeed, impairment of the choroidal circulation leads to a reduction of the intraretinal vascular flow and, invariably, to retinal photoreceptor damage. Hence, the use of parameters that evaluate structural and vascular choroidal alterations as potential biomarkers of retinal damage. The introduction of the latest techniques of SDOCT, in particular, of EDI, have enabled the non-invasive study of the choroid that is more accessible and immediate. Recently, the binarization technique has enabled the evaluation of the CVI, a parameter that combines both a structural and vascular study of the choroid. This overcomes the limitations of CT calculation that can be affected by systemic and ocular confounding factors such as age and axial length. While there is no consensus on CT changes in patients with RP, probably in relation to its variability, the CVI is always reduced implying that this can be a reliable and valid marker in assessing the progression and manifestation of RP. Moreover, CVI may also represent a valid marker in the evaluation of retinal complications such as CMO in RP patients.

In conclusion, based on the studies currently available, CVI, possibly integrated with the CT, may be considered a valid biomarker of damage in the progression of RP. However, further studies are warranted to better characterize the evolution of disease in different scenarios of retinal severity and impairment. This could be a valid starting point not only on a diagnostic but also at a therapeutic level since a better understanding of the role of the choroid in RP may suggest alternative therapeutic strategies or be used as a biomarker/clinical endpoint for potential therapies.

### Summary

#### What is known about this topic

- In retinitis pigmentosa, choroidal alterations have a role in the pathogenetic mechanism that leads to photoreceptor damage.
- Choroidal haemodynamics studies have shown that there is an alteration of retinal and choroidal vascularization in retinitis pigmentosa.

#### What this study adds

- In vivo imaging with spectral domain optical coeherence tomography shows choroidal thickness changes in retinitis pigmentosa but consensus is lacking owing to confounding factors such as age.
- Choroidal vascularity index is reduced in retinitis pigmentosa and this parameter is a reliable and valid marker in assessing retinitis pigmentosa.
